# Recent Advances in Carbon Dots for *In Vitro/Vivo* Fluorescent Bioimaging: A Mini-Review

**DOI:** 10.3389/fchem.2022.905475

**Published:** 2022-05-05

**Authors:** Chen He, Xiaofeng Lin, Yanqiu Mei, Yan Luo, Min Yang, Ying Kuang, Xiaoqing Yi, Weijia Zeng, Qitong Huang, Bin Zhong

**Affiliations:** ^1^ Department of Pharmacy, First Affiliated Hospital of Gannan Medical University, Ganzhou, China; ^2^ School of Pharmacy, Gannan Medical University, Ganzhou, China; ^3^ Key Laboratory of Biomedical Sensors of Ganzhou, Ganzhou Key Laboratory of Immunotherapeutic Drugs Developing for Childhood Leukemia, Key Laboratory of Biomaterials and Biofabrication in Tissue Engineering of Jiangxi Province, School of Medical and Information Engineering, School of Public Health and Health Management, Oil-Tea in Medical Health Care and Functional Product Development Engineering Research Center in Jiangxi, Gannan Medical University, Ganzhou, China

**Keywords:** *in vitro* bioimaging, *in vivo* bioimaging, fluorescence imaging, nanomedicine, carbon dots

## Abstract

As a new type of “zero-dimensional” fluorescent carbon nanomaterials, carbon dots (CDs) have some unique optical and chemical properties, they are being explored for a variety of applications in bio-related fields, such as bioimaging, biosensors, and therapy. This review mainly summarizes the recent progress of CDs in bioimaging. The overview of this review can be roughly divided into two categories: (1) *In vitro* bioimaging based on CDs in different cells and important organelles. (2) The distribution, imaging and application of CDs in mice and zebrafish. In addition, this review also points out the potential advantages and future development directions of CDs for bioimaging, which may promote the development of CDs in the field of bioimaging.

## 1 Introduction

Biological imaging covers many modalities, including X-ray, B-ultrasound, Computed tomography (CT), Positron emission computed tomography (PET), Magnetic resonance imaging (MRI), and so on (Li et al., 2019; Molkenova et al., 2019; Molkenova et al., 2020; Wen et al., 2020; Mulikova et al., 2021; Olifirenko et al., 2021; Yi et al., 2022). Among them, fluorescence imaging technology plays an important role in bioimaging due to its advantages of high sensitivity, easy observation and simple instrument. At present, many fluorescent material have been used for biological imaging, such as organic small molecules (Wu J. et al., 2021), nanomaterials (Deling Li et al., 2020), and so on. Since carbon dots (CDs) were synthesized in 2004 (Xu et al., 2004), they have received extensive attention in various fields as a new type of fluorescent probe. Because of their multicolor luminescence (Kailasa et al., 2019; Jiao et al., 2020; Ghosh et al., 2021a; Ghosh et al., 2021b), tunable optical properties (Wang et al., 2020a), superior chemical and photostability (Wang et al., 2020b; Rao et al., 2020), low cytotoxicity and excellent biocompatibility (Huang et al., 2020; Kuang et al., 2020; Lin et al., 2021a; Lin et al., 2021b; Mei et al., 2022), CDs are promising candidates bioimaging. Due to the easy functionalization and good biocompatibility of the surface of CDs, they can also be used as an effective tool for visual monitoring of biological processes (Sun et al., 2021; Vedhanayagam et al., 2021; Huang et al., 2022). More importantly, the synthesis method of CDs is simple, environmentally friendly, economical, and energy-saving, and their synthetic raw materials are widely sourced, green and cheap. Therefore, a wide range of CDs preparation methods and sources of raw materials provide opportunities to achieve biological *in vitro* and *in vivo* imaging.

In recent years, several reviews have been summarized on applications of CDs for neurological treatment, biosensors, photocatalysis, bioimaging, and so on (Ashrafizadeh et al., 2020; Younis et al., 2020; Kailasa and Koduru, 2022; Wang and Lu, 2022; Ðorđević et al., 2022). At present, the review on the bioimaging of CDs mostly focuses on the *in vitro* bioimaging of CDs, such as imaging of cancer cells and nerve cells, etc. In this review, we survey the latest research on the application of CDs in the rapidly evolving bioimaging field, especially *in vitro* bioimaging of important organelles (nucleus, mitochondria) and *in vivo* bioimaging (mice, zebrafish). This review aims to provide readers with the latest, most exciting and influential research in this field. What’s more, the opportunities and challenges of carbon dots in bioimaging applications in the future are briefly discussed. Eventually, we hope to provide some new ideas for developing bioimaging based on CDs.

## 2 *In Vitro* Bioimaging

CDs have shown excellent potential in the field of bioimaging due to the CDs have good dispersibility, the ability to specifically bind to target units, high near-infrared absorption and photoluminescence efficiency, good chemical stability and photostability (Bondon et al., 2020; Lin et al., 2020; Cardoso Dos Santos et al., 2020; Wang et al., 2021). As known, Different types of cells have different structures and morphologies, and different cell membranes or cytoplasm contain different biomarkers, leading to specific responses to foreign nanoparticles. On this basis, CDs with special functions were synthesized and used in biological imaging technology, which also provided the possibility of applying CDs *in vivo* bioimaging, which hope to promote the development of cell imaging techniques.

### 2.1 Cell Bioimaging

#### 2.1.1 Cancer Cells Bioimaging

**FIGURE 1:**
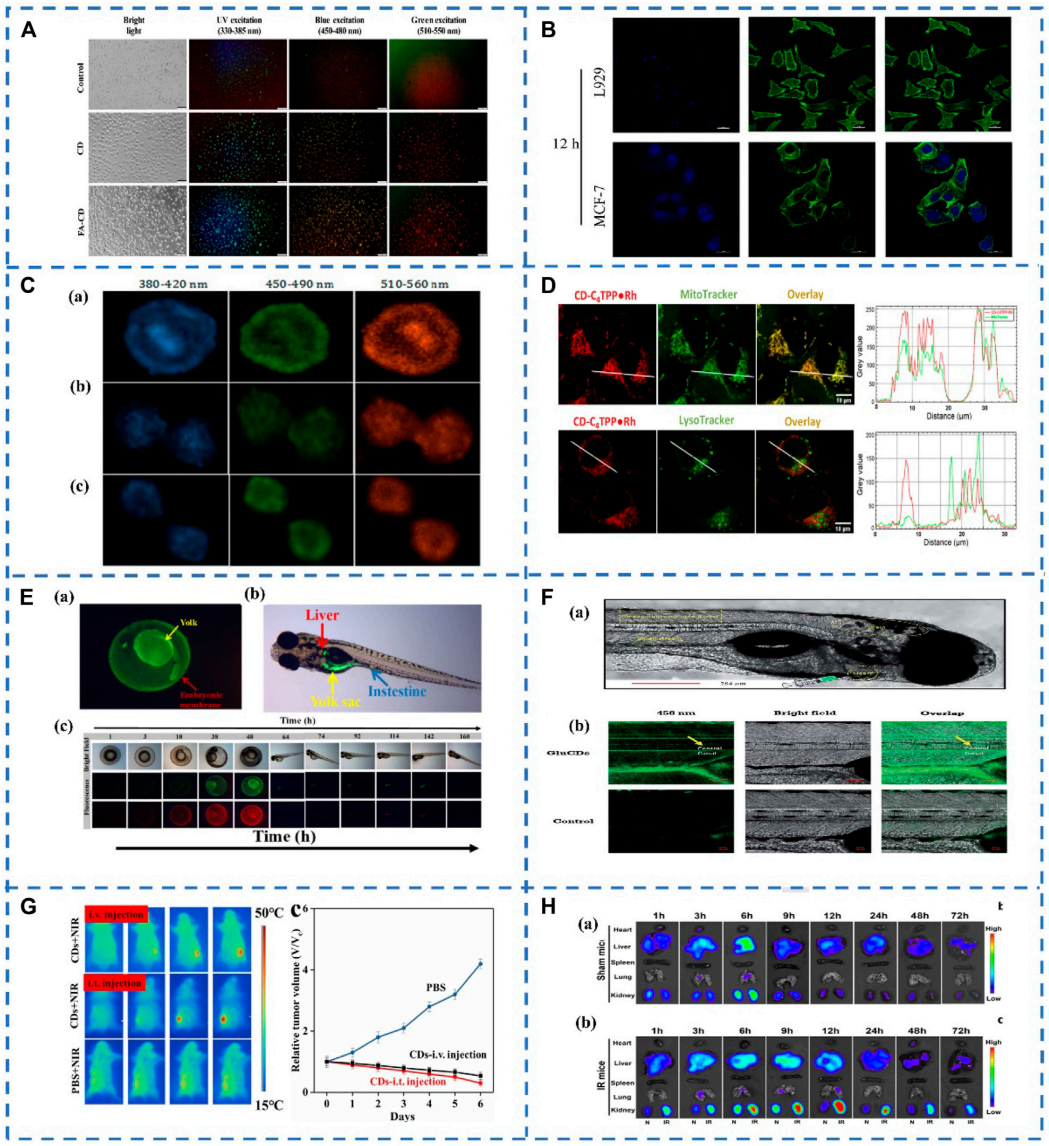
**(A)** Bioimaging of CDs and FA-CDs in cancer cells (Gudimella et al., 2021). **(B)** Flow cytometry profiles of MCF-7 cells and L929 cells treated with the CDs-PEI-AS1411 for 12 h (Kong et al., 2020). **(C)** Mitotic cells stained by N-CDs visualizing chromosome aggregation through progression of mitosis’ prophase, metaphase and anaphase, respectively (Zhang et al., 2020). **(D)** N-CDs internalization in live MDA-MB-231 cells. (Kaminari et al., 2021); **(E) (a)** Uptake and distribution of WCDs in zebrafish embryos; **(b)** The distribution of WCDs in zebrafish larvae; **(c)** Fluorescence microscopy images of uptake of WCDs in zebrafish growth cycle (Zonglin Liu et al., 2021). **(F) (a)** Confocal images of wild-type zebrafish showing the injection route, heart, blood stream, CNS and observation area (central canal of spinal cord). **(b)** Accumulation of GluCDs-F in the CNS of zebrafish (Seven et al., 2021). **(G)** Representative infrared thermal images of tumor-bearing mice with intravenous injection, intratumoral (i.t.) injection of CDs (50 μl, 20 mg/ml) and PBS (50 μl) into the tumor site under 808 nm laser irradiation and time course change in the relative volume after treatment by using CDs and PBS (Li et al., 2019) **(H)** *In vivo* bio-distribution of PDA-CDs in both ischemia-reperfusion (IR)-AKI and healthy mice.Representative fluorescence images (excitation: 410 nm, emission: 500 nm) of major organs from shammice **(a)** and IR-injured mice **(b)** at different times after PDA-CDs intravenous injection (Gao et al., 2020).

Cancer is one of the greatest challenges facing humanity today, and many people die from cancer every year, early diagnosis of cancer and prevention of malignant tumors are extremely important. Currently, CDs can penetrate a variety of cancer cells, what’s more due to the stable optical properties and excellent biocompatibility of CDs, cancer cells can be detected by fluorescence imaging of CDs (Mchugh et al., 2018). Gudimella et al. synthesis of fluorescent CDs from the citrus peel as a renewable green resource, and then the CDs was conjugated with folic acid (FA-CDs). As shown in Figure 1A, MCF-7 cells treated with FA-CDs had brighter fluorescence emission than MCF-7 cells treated with CDs, indicating that FA-CDs are a remarkable material for cell imaging (Gudimella et al., 2021). Different CDs can specifically recognize cancer cells by interacting with groups on the surface of cancer cells. However, cellular uptake of free CDs lacks selectivity, and the same negative charge as the cell membrane may lead to inefficient cellular internalization. Based on the modification of CDs with the DNA aptamer AS1411 with polyimine (PEI) as the bridge. Kong et al. developed a surface charge inversion nanosystem, using the DNA aptamer AS1411 labeled CDs nanoparticle probe for specifically targeted bioimaging of cancer cells (Figure 1B) (Kong et al., 2020). In addition, Mahani et al. demonstrated the selective targeting and imaging of hepatoma cells by fluorescent CDs molecularly imprinted polymers (CDs-MIPs). The overexpression of monosaccharides on cancer cells can act as targeting molecules. On these cells, CDs-MIP specifically binds to D-glucuronicacid (GlcA) and N-acetylneuraminicacid (NANA), resulting in high-contrast images in cancer cells imaging (Mahani et al., 2021).

#### 2.1.2 Neural Cells Bioimaging

An in-depth and comprehensive understanding of the nature and function of the nervous system can lead to more effective treatment of brain diseases. Therefore, it is necessary to develop a new brain bioimaging method to visualize the relationship between brain structure, neuronal activity and neurochemistry. Due to their excellent biocompatibility, stable fluorescence properties of CDs can help to overcome current challenges in neuroimaging. Pei et al. used lignin as the starting carbon source to synthesize carbon quantum dots CDs by hydrothermal method. The team explored the imaging of lignin CDs in N27 cells (a rat neural cell line used as a model of dopaminergic neurons). A large accumulation of CDs was observed in the cytoplasm and nucleus, which indicated that CDs have good permeability of cell membrane and other intracellular organelle membranes. It could be applied to biological imaging of brain cells in the future (Pei et al., 2021). Besides. Wu et al. developed a novel two-photon fluorescent probe M9, which consists of graphene oxide (GO), red-emitting CDs, and azobenzene bound to DNA (DNA-Azo). For *in situ* imaging of miR-9 in living neurons and brain tissue of AD mice. It was found that the M9 fluorescent probe easily entered neuron cells and distributed them in the cytoplasm of neuron cells. These results shed light on understanding the genetic basis of AD and hold great promise for the early diagnosis of cancer and neurological disorders (Wu et al., 2020). In conclusion, due to the penetrability and good biocompatibility of CDs, they can provide a good possibility for developing effective methods for clinical diagnosis and treatment of the central nervous system.

### 2.2 Organelles Bioimaging

Nowadays, fluorescence imaging technology is becoming more and more mature, and more and more fluorescent probes for biological imaging have been developed, but the bioimaging of subcellular and organelles still needs further efforts to track changes in cell morphology or function. Biomaging of cell nuclei is crucial for revealing nuclear morphology and its role in cell metabolism, growth, differentiation, and inheritance (Zhou et al., 2020). Zhang et al. synthesized nitrogen doped CDs (N-CDs) by using citric acid as raw material and propylene diamine as a passivation agent. Then the N-CDs were used for HeLa cell staining, the results showed that N-CDs were located in the nucleus, showing a multicolor luminescence effect, and there was almost no blue-green-red fluorescence intensity in the cytoplasm (Figure 1C). The N-CDs will be automatically enriched in the nucleus, and the concentration in the cytoplasm is very low, and they could be a new way to track individual cells or visualize processes such as nuclear marker-based mitosis, which may provide new tools for tracking chromatin phase changes during cell cycle changes (Zhang et al., 2020).

Mitochondria are one of the most important subcellular compartments in the cell, the site of ATP production and the center of cellular metabolism. The state of mitochondria is associated with a variety of diseases, grasping the state of mitochondria plays a crucial role in the treatment of various diseases (Anqi Li et al., 2020; Mani et al., 2021). CDs can be a new mitochondrial imaging probe, a series of N-CDs were prepared by Kaminari et al. The N-CDs exhibit subcellular mitochondrial localization and compared to known mitochondrial probes, multi-functionalized N-CDs exhibit superior photostability, stable long-term mitochondrial imaging, and cell compatibility with apoptotic labeling potential (Figure 1D). Their uptake depends on mitochondrial membrane potential and induces their preferential localization in malignant cells, which is expected to serve as a carrier for mitochondria-targeted delivery of anticancer drugs (Kaminari et al., 2021). Guo et al. synthesized fluorescent CDs that could observe cell viability *in situ* by simple microwave-assisted synthesis. Due to electrostatic interactions, positively charged CDs tend to accumulate in mitochondria with high negative MMPs in healthy cells. When cells are damaged with a concomitant decrease in MMP levels, CDs migrate from the mitochondria to the nucleolus due to their binding affinity to nucleic acids. Once the cellular state is restored, MMP levels are again highly reduced, and the CDs are reversed back to the mitochondria. Therefore, the viability of cells can be easily observed through the different spatiotemporal distribution of CDs in living cells. The discovery of CDs has great potential in the study of cell survival (Guo et al., 2021).

The *in vitro* bioimaging demonstrate that CDs have broad application prospects in the biomedical field. The CDs can be used as fluorescent probes for cancer cell imaging and organelle targeting for the diagnosis and prevention of some major diseases. However, the current CDs-based fluorescent probes still have some defects, we should focus on developing fluorescent probes with better performance, such as precise targeting ability, high fluorescence quantum yield, high stability, simple surface functionalization, and so on. In addition, the targeting of CDs and the application of *in vivo* bioimaging should be further investigated in detail.

## 3 *In Vivo* Bioimaging

In recent years, *in vivo* biomedical applications based on fluorescent CDs have made many efforts for future clinical diagnosis and therapy. Yang et al. (2009) are the first to report *in vivo* imaging of CDs in mice via three injection routes. Since then, more and more animal models have been established to explore the imaging studies of CDs *in vivo*.

### 3.1 Biodistribution of CDs in Zebrafish and Mice

CDs play an important role in the medical field. *In vivo* imaging of CD may have chronic toxicity, low stability, and potential for accumulation. Therefore, the distribution and metabolism of CD *in vivo* should be assessed. Liu et al. synthesized *F.nucleatum*-CDs (Fn-CDs) for *in vivo* imaging in mice. The CDs were injected into mice and detected Fn-CDs in mice at different periods. At different periods, different parts of the mice appeared fluorescence successively. After that, the fluorescence intensity of Fn-CDs gradually decreased, indicating that Fn-CDs may have entered the blood circulation. What’s more, the fluorescence disappeared after 24 h, which manifesting that Fn-CDs can be excreted through digestive system metabolism, making Fn-CDs an excellent candidate for *in vivo* bioimaging and biosensing (Lijuan Liu et al., 2021). Besides, Liu et al. synthesized high-yield water-soluble CDs (WCDs) by the targeted method with good biocompatibility. The uptake and metabolism of WCDS *in vivo* were studied by zebrafish. When WCDs were cultured with zebrafish embryos, WCDs mainly existed in the yolk of the zebrafish embryonic stage. In adult zebrafish, green fluorescence is distributed in the intestine, stomach, liver and yolk sac (Figure 1E). By observing the fluorescence imaging of WCDs in zebrafish at different periods, the distribution and metabolism of WCDs in zebrafish can be observed (Zonglin Liu et al., 2021). These research works provide valuable information for the administration of CDs *in vivo*, monitoring the therapeutic effect, etc. By observing the distribution and metabolism of CDs *in vivo*, evaluating the toxicity of CDs *in vivo*, and reducing the potential cellularity of CDs through surface modification.

### 3.2 Fluorescent Bioimaging-Guided Drug Delivery System

The blood-brain barrier (BBB) is one of the most important factors limiting the development of treatments for neurological diseases and brain cancer. Many drugs cannot directly penetrate the BBB, resulting in very limited drug delivery systems (DDS) for the treatment of central nervous system-related diseases and brain cancer. Therefore, the development of new DDS is very necessary. CDs-mediated DDS has received extensive attention due to its penetration of the blood-brain barrier (Liyanage et al., 2020). A fluorescein carbon dots prepared from glucose (GluCDs-F) was synthesized by Seven et al. They tested the ability of GluCDs-F to cross the blood-brain barrier in zebrafish and rat models. After intravenous administration in rats, GluCDs-F was observed to concentrate in cervical spinal cord gray matter (e.g., ventral horn, dorsal horn, mid-gray) in the central nervous system, consistent with aggregation behavior in neurons. Therefore, GluCDs-F-targeted neurons have great potential as a drug delivery platform in neurodegenerative diseases, traumatic injuries, and central nervous system malignancies (Figure 1F). In addition, to enhance tumor-specific imaging and drug delivery, tumor drug molecule delivery systems can be realized by targeting probe-binding ligands that recognize receptor-like molecules (Seven et al., 2021). Li et al. reported large amino acid mimetic CDs (LAAM-CDs) for selective imaging and drug delivery to tumors, including brain tumors. The LAAM-CDs were used as a DDS utilizes specific carrier transporters that are differentially upregulated in cancer cells. LAAMTC-CDs, a type of LAAM-CDs, which were synthesized by using 1,4,5,8-tetraaminoanthraquinone (TAAQ) and citric acid (CA). Then LAAMTC-CDs were administered intravenously to patients with U87 glioma in mice and analyzed using near-infrared fluorescence imaging at different time points. The accumulation of LAAM TC-CDs in the brain increased over time by fluorescence imaging profiles and peaked at 8–12 h after injection when the fluorescence signal in the brain dominated. Therefore, LAAM-CDs had the potential to translate into clinical applications for imaging and drug delivery in various tumors and diseases of the central nervous system (Shuhua Li et al., 2020).

### 3.3 Fluorescent Bioimaging Guides the Treatment of Cancer and Kidney Disease

Cancer treatment is one of the biggest challenges facing the medical field today. Li et al. have explored watermelon-derived CDs with secondary near-infrared (NIR-II) emission as *in vivo* optical fluorescent agents, which in addition to their excellent optical properties, also possess excellent 808 nm laser-induced photothermal properties, photothermal therapy (PTT) for cancer and CD has a good biological function (Figure 1G). These studies verified the potential application of CDs in the treatment of cancer PTT (Li et al., 2019). Due to insufficient light-to-heat conversion, developing an ideal CDs is still difficult, requiring the aid of high-power-density lasers. Therefore, Kim et al. prepared sulfur-doped CDs (S-CDs) with strong near-infrared absorption ability using *Japanese camellia* as raw material by hydrothermal method. And the lower dose of S-CDs was found to have higher PTT performance (Kim et al., 2021). Acute kidney injury (AKI) is a reactive oxygen species (ROS)-promoted disease with high mortality and morbidity for which there is currently no effective drug treatment. Gao et al. prepared m-phenylenediamine-based CDs (PDA-CDs) with ultra-small-sized glomerular filtration barrier permeability and antioxidant properties, the PDA-CDs exhibited significant ROS scavenging *in vitro* and *in vivo* active. Both AKI mice and healthy mice were injected with PDA-CDs intravenously. Then major organs (heart, liver, spleen, lung, and kidney) were taken for fluorescent bioimaging of fluorescence-specific parameters of PDA-CDs. Normal mouse PDA-CDs mainly accumulated in the liver and kidney. In AKI mice, PDA-CDs mainly accumulated in the liver and kidney (Figure 1H), and after injection of 12 h, the accumulation of PDA-CDs in the kidneys of AKI mice reached a peak, which was much longer than that in normal kidneys. Meanwhile, by comparing with the cisplatin-induced AKI model, it was found that PDA-CDs exhibited significant therapeutic effects in both models. This provides an effective drug therapy strategy for ROS-induced AKI with significant clinical translation potential (Gao et al., 2020).

In a short, *in vivo* bioimaging based on CDs have broad application, the above work validates the ability of CDs to have potent chemotherapeutic effects, which can provide a strategy for the potential clinical application of CDs in image-guided tumor therapy, renal disease treatment and other diseases.

## 4 Conclusion and Future Prospects

Till now, CDs have already proven to be an intriguing class of nanoparticles, which have made significant achievements in the field of bioimaging due to their excellent fluorescence properties, good biocompatibility, low toxicity, high sensitivity, and easy surface functionalization. This review mainly summarizes recent advances in the field of CDs-based bioimaging in two aspects (Table 1): *in vitro* bioimaging of important organelles (nucleus, mitochondria) and *in vivo* bioimaging (mice, zebrafish). Although the CDs have shown many advantages in bioimaging, there are still some challenges: (1) How to synthesize CDs with excellent fluorescent properties by a simple synthesis method and explain the synthesis mechanism? (2) More experimental and theoretical studies should be carried out to elucidate the fluorescent mechanism of the CDs. (3) CDs with red or near-IR fluorescent emission are more suitable for bioimaging *in vitro/vivo*, it is very important to prepare CDs with red or near-IR fluorescent emission which have high quantum yield and excellent bioimaging performance. (4) With the advancement of bioimaging methods and equipment, efficient tissue penetration and *in vivo* bioimaging under low background fluorescence will continue to be achieved, which will further promote the development of CDs in bioimaging. We believe that with the continuous development of the field, the practical application of CDs in bioimaging will be greatly improved.

**TABLE 1:** Application of different types of carbon dots in bioimaging.

Precursor of CDs	Materials of bioimaging	Application in bioimaging	References
Citrus fruit peels	FA-CDs	MCF-7 cells imaging	Gudimella et al. (2021)
Citric acid (CA)	CDs-polyethyleneimine-AS1411	MCF-7 and L929 cells imaging	Kong et al. (2020)
Tinospora cordifolia leaves	CDs	B_16_F_10_ Melanoma and SiHa cervical cancer cells imaging	Mohapatra et al. (2022)
CA	CD-MIPs	MCF-7, HepG-2, and NIH-3T3 cells imaging	Mahani et al. (2021)
Fresh tea leaves + urea	N-CDs	A549 cells imaging	Ge et al. (2022)
Formamide + Phosphoric acid	N, P-CDs	HeLa cell imaging	Naixin Li.et al. (2021)
Sulfonated tetraphenylporphyrin	CDs	HeLa cell imaging	Li-ping Li.et al. (2021)
Rose bengal+1,4-dimercaptobenzene	S-CDs	HPAEpiCs and A549 cells imaging	Yu et al. (2022)
Kiwi fruit peel	CDs	imaging human normal and cancer cells	Atchudan et al. (2022b)
Betel leaves	CDs	Mouse fibroblast L929 cells imaging	Atchudan et al. (2022a)
Lignin	CDs	Neuronal N27 cells imaging	Pei et al. (2021)
Thiourea + o-PDA	CDs-DNA-Azo	miR-9 imaging in AD mouse brain tissue	Wu et al. (2020)
CA + Propylene diamine	N-CDs	HeLa cells nuclear chromatin imaging	Zhang et al. (2020)
Propylene Glycol + Protamine	Protamine-CDs	HEK-293 cells nucleus imaging	Zhang et al. (2021)
p-PDA+4-formylbenzeneboronic acid	CDs	RAW 264.7 murine cells line nucleus imaging	Phukan et al. (2022)
CA + Ethylenediamine	CDs	A-MB-231 cells mitochondria imaging	Kaminari et al. (2021)
CA + p-PDA	CDs	LO-2 cells and Hep3B cells imaging	Jin et al. (2022)
Metformin	CDs	Mitochondrial imaging and targeting capabilities	Cilingir et al. (2021)
CA + N,N-dimethylaniline	CDs	Image mitochondria in cells and observe cell viability	Guo et al. (2021)
F. nucleatum	Fn-CDs	*In vivo* fluorescence imaging of male Kunming mice	Lijuan Liu et al. (2021)
Carrots + Acrylamide	WCDs	Imaging, uptake and distribution of WCDs in zebrafish	Zonglin Liu et al. (2021)
M-PPD+1,2,3-propanetricarboxylic acid	N-CDs	Imaging exogenous ClO^−^ in cell nucleus and living zebrafishes	Wu et al. (2021b)
Glucose	GluCD-F	Zebrafish *in vivo* imaging and drug delivery system	Seven et al. (2021)
1,4,5,8-tetraminoanthraquinone + CA	CDs	*In vivo* imaging and drug delivery system in U87 glioma mice	Shuhua Li et al. (2020)
Panax notoginseng	N-CDs	As diagnostic tools and contrast dyes for biomedical applications	Zheng.et al. (2021)
Caulis polygoni multiflora	CDs	*In vivo* imaging in mice and as a disease detection tool in physiology and pathology	Chang et al. (2021)
Camellia japonica	S-CDs	*In vivo* tumor imaging and photothermal therapy of cancer in HT-29 tumor-bearing mice	Kim et al. (2021)
Trf + Glucose oxidase	Iron-doped CDs	C6-LUC cell imaging and targeted therapy for the treatment of gliomas	Liu et al. (2022)
Hematoporphyrin	HP-CQDs	Breast cancer cells (MCF-7) imaging and Photodynamic therapy aids in clearing breast cancer cells	Murali et al. (2022)
m-PDA	m-PDA-based CDs	*In vivo* imaging and mitigation of acute kidney injury in IR-AKI mice	Gao et al. (2020)
Cucumis melo	CDs	Cunninghamella elegans cells, Aspergillus flavus cells, and Rhizoctonia solani cells imaging	Desai et al. (2019)
